# MiR-450a-5p strengthens the drug sensitivity of gefitinib in glioma chemotherapy via regulating autophagy by targeting EGFR

**DOI:** 10.1038/s41388-020-01422-9

**Published:** 2020-08-20

**Authors:** Yu Liu, Liang Yang, Fan Liao, Wei Wang, Zhi-Fei Wang

**Affiliations:** 1 Department of Neurosurgery, The Third Xiangya Hospital of Central South University, Changsha, 410013 P.R. China; 2 Department of Neurology, The Second Xiangya Hospital of Central South University, Changsha, 410011 P.R. China

**Keywords:** Molecular biology, Stem cells

## Abstract

Glioma reported to be refractory to EGFR tyrosine kinase inhibitor is the most common malignant tumor in central nervous system. Our research showed the low expression of miR-450a-5p and high expression of EGFR in glioma tissues. MiR-450a-5p was also observed to synergize with gefitinib to inhibit the proliferation, migration and invasion and induce the apoptosis and autophagy of glioma cells. Furthermore, miR-450a-5p was demonstrated to target 3′UTR of EGFR, and regulated EGFR-induced PI3K/AKT/mTOR signaling pathway. Moreover, the above effects induced by miR-450a-5p in glioma cells were reversed by WIPI1 silencing. The inhibition role of miR-450a-5p on glioma growth was also confirmed in vivo by subcutaneous and intracranial tumor xenografts. Therefore, we conclude that miR-450a-5p synergizes with gefitinib to inhibit the glioma tumorigenesis through inducing autophagy by regulating the EGFR-induced PI3K/AKT/mTOR signaling pathway, thereby enhancing the drug sensitivity of gefitinib.

## Introduction

Glioma is a primary malignant brain tumor commonly seen in the central nervous system, characterized by rapid disease progression, significant individual difference, and poor prognosis [1]. The investigations have uncovered that glioblastoma chemoresistance is an independent factor affecting the prognosis of patients [2, 3]. Due to the deepening understanding of the molecular biology and genetic characteristic of malignant glioma, targeted therapy against glioma is gradually becoming possible [4].

Epidermal growth factor (EGFR) is a kind of transmembrane glycoprotein and expressed in cells of normal epithelial-derived tissues, and overexpressed in many human tumors [5]. There are many studies using EGFR as the therapeutic target, among which tyrosine kinase inhibitors (TKI) are the most successful ones [6]. Gefitinib is a small molecule inhibitor of EGFR tyrosine kinase, and it can inhibit receptor phosphorylation and block the subsequent activation of EGFR-mediated signaling pathways [7]. Gefitinib has been applied for clinical application in non-small cell lung cancer (NSCLC), and exhibited anti-proliferative and anti-invasive effects in glioblastoma in vitro [8–10]. However, gliomas were refractory to TKI and EGFR-associated TKI only showed marginal benefits in recurrent malignant gliomas [11].

MicroRNA (miRNA) is a kind of non-coding small RNA and specifically binds to the 3′untranslated region (3′-UTR) of the target mRNA and regulates gene expression at the posttranscriptional level [12]. Previous studies have shown that miRNAs are involved in biological processes including tumor apoptosis, proliferation, differentiation, metastasis, angiogenesis, immune response, etc [13, 14]. MiR-450a is an intragenenic miRNA located on chromosomal location Xq26.3. In recent years, through next-generation sequencing and miRNA microarray, studies revealed that miR-450a-5p had frequently abnormal expression in various of cancers and affected the tumorigenesis of malignant tumors [15, 16]. However, the effects of miR-450a-5p in gliomas are still unknown. Our preliminary study showed that miR-450a-5p was differentially expressed in gliomas, and was predicted to have binding sites on EGFR. But, the effects of miR-450a-5p in gliomas and the roles of miR-450a-5p in the regulation of EGFR are still unknown. MiRNAs associated with IGF-1R signaling pathway were involved in the resistance against EGFR-TKIs [17, 18]. Therefore, the question of whether differential expression of miR-450a-5p in gliomas is related to the sensitivity of gefitinib is investigated in this study.

Autophagy is an evolutionarily conserved process that degrades damaged organelles and promotes the metabolism of proteins to maintain cellular homeostasis [19]. Autophagy is initiated and executed by autophagy related (ATG) proteins to form autophagosomes [20]. The human WD-repeat protein interacting with phosphoinositides (WIPI) family proteins play critical roles in recognizing the PtdIns3P enriched on autophagosomes, functioning as autophagy-specific PtdIns3P-binding effectors [20, 21]. Studies showed that EGFR regulated autophagy through multiple pathways [22]. Therefore, regulating the autophagy level will be a feasible way to improve the therapeutic effect of EGFR inhibitors in cancers [23]. The PI3K/Akt/mammalian target of rapamycin (mTOR) pathway is a major regulatory pathway of autophagy, in which PI3K can be phosphorylated by EGFR via receptor tyrosine activity [19, 24].

In our study, we demonstrated that miR-450a-5p regulated the autophagy occurrence in glioma by targeting EGFR-mediated PI3K/AKT/mTOR pathway, thereby regulating the proliferation, apoptosis, and migration of glioma cells and enhancing the sensitivity of glioma cells to gefitinib. In addition, we firstly found that WIPI1 knockdown reversed the above effects induced by miR-450a-5p via blocking the progression of autophagy. With these findings, miR-450a-5p and WIPI genes or proteins might present novel targets to overcome gefitinib resistance in glioma treatment.

## Results

### MiR-450a-5p showed a negative correlation with the metastasis of glioma

**Fig. 1:**
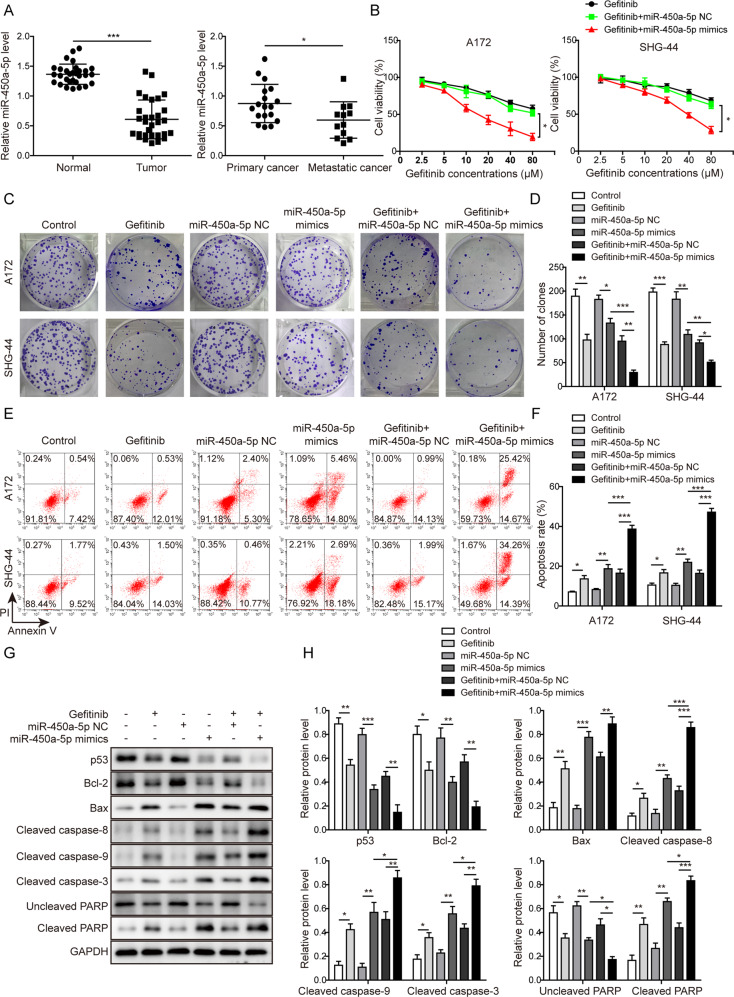
MiR-450a-5p synergizes with gefitinib to impact the glioma cells proliferation and apoptosis. **a** The expression of miR-450a-5p in 30 pairs of glioma samples (18 cases of primary glioma samples and 12 cases of metastatic glioma samples) and corresponding normal tissues detected by qRT-PCR. **b** Effect of miR-450a-5p and gefitinib on the cell proliferation of A172 and SHG-44 cells detected by the MTT assay. **c** The cell proliferation after different treatments determined by the colony formation assay. **d** Quantitative analysis of the numbers of colonies. **e** Effect of miR-450a-5p and gefitinib on the cell apoptosis investigated by flow cytometry. **f** Quantitative analysis of apoptosis rate. **g** Expression levels of indicated cell apoptosis-related proteins detected by Western blot analysis. **h** Quantitative analysis of the Western blot results. The transfected cells were treated with gefitinib (20 μM for A172 cells and 40 μM for SHG-44 cells). In the colony formation assay, the cells were cultured for 2 more weeks. In all other assays, the transfected cells were cultured for 48 h. The result was a representative of three independent experiments. Note: Error bars represented mean ± SD. *p* values were determined by one-way analysis of variance (ANOVA) followed by Tukey post hoc test. ***p < 0.001, **p < 0.01 and *p < 0.05.

As shown in Fig. 1a, the expression of miR-450a-5p in 30 tumor tissues was significantly lower than that in corresponding normal tissues, especially in 12 cases of metastatic glioma samples. Clinicopathological characteristics of patients were summarized in Table S1 and showed the miR-450a-5p expression was strongly associated with the KPS scores and WHO grades of the patients (*P* < 0.05), decreased dramatically in patients with >80 KPS scores and tumor stages of III–IV. These data indicate that miR-450a-5p expression might have a negative correlation with the metastasis of glioma.

### MiR-450a-5p synergizes with gefitinib to impact the glioma cells proliferation and apoptosis

Then, as shown in Fig. 1b, the cell viability of both cells was decreased after gefitinib treatment in a concentration-dependent manner. The IC_50_ of gefitinib for A172 and SHG-44 cells were 56.15 μM and 81.56 μM, respectively. Overexpression of miR-450a-5p was observed to further reduce the cell viability under the same concentration of gefitinib with an IC_50_ value of 25.02 μM and 48.59 μM, respectively. These indicate that overexpression of miR-450a-5p might downregulate the IC_50_ values of gefitinib and enhance the drug sensitivity to glioma cells. For the following experiments, 20 μM and 40 μM gefitinib were used for treating A172 and SHG-44 cells, respectively.

As shown in Fig. 1c, d, much fewer colonies were observed after treatment with gefitinib or miR-450a-5p mimics, and were further inhibited with the co-treatment. However, this inhibition caused by gefitinib was moderately reversed by downregulating miR-450a-5p (Fig. S1a–b). Moreover, gefitinib treatment or miR-450a-5p mimics transfection promoted the cell apoptosis, which was further induced after miR-450a-5p overexpression in gefitinib-treated cells (Fig. 1e, f). As expected, this induction was partly reversed by downregulating miR-450a-5p (Fig. S1c–d). A similar pattern was observed as shown in Fig. 1g, h and Fig. S1e–f, in which the expression levels of Bcl-2, p53, and uncleaved PARP were decreased, while Bax, cleaved caspase-8, cleaved caspase-9, cleaved caspase-3, and cleaved PARP were dramatically increased in the gefitinib-treated cells. Also, miR-450a-5p overexpression was observed to strengthen the effects of gefitinib, while miR-450a-5p downregulation showed a reversed effect. All these results indicate that miR-450a-5p may synergize with gefitinib to inhibit the glioma cell proliferation and induce cell apoptosis.

Also, miR-450a-5p overexpression significantly promoted the cell apoptosis while downregulation of miR-450a-5p exerted the inverse effect on the apoptosis of normal glial cells (see Supplementary Results section and Supplemental Fig. S2 for further details). In addition, combination treatment of miR-450-5p overexpression and gefitinib also showed promotive effects on apoptosis of normal glial cells (see Supplementary Results section and Supplemental Fig. S3 for further details).

### MiR-450a-5p synergizes with gefitinib to impact the glioma cells migration and invasion

**Fig. 2:**
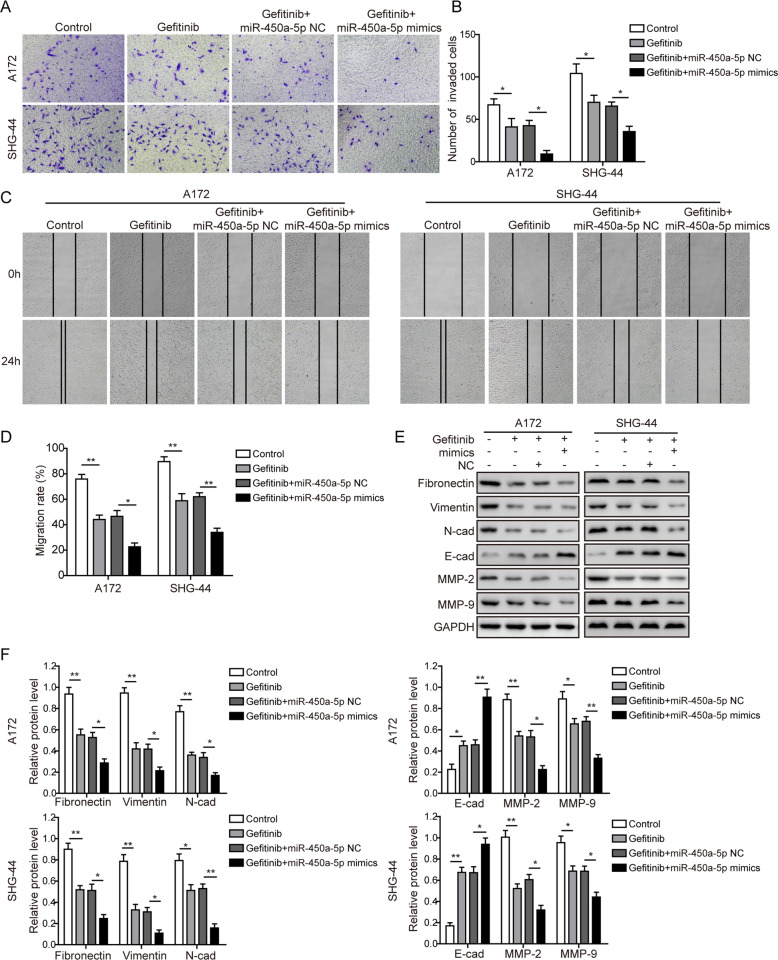
MiR-450a-5p synergizes with gefitinib to impact the glioma cells migration and invasion. **a** The cell invasion abilities of glioma cells detected by the Transwell invasion assay. **b** Quantitative analysis of the invaded cells. **c** The cell migration abilities of glioma cells detected by the wound healing assay. **d** Quantitative analysis of the migration rate. **e** Expression levels of indicated EMT-related proteins detected by Western blot analysis. **f** Quantitative analysis of the Western blot results. In all assays, the transfected cells were cultured with DMEM medium containing gefitinib (20 μM for A172 cells and 40 μM for SHG-44 cells) for 48 h. The result was a representative of three independent experiments. Note: Error bars represented mean ± SD. *p* values were determined by one-way analysis of variance (ANOVA) followed by Tukey post hoc test. **p < 0.01 and *p < 0.05.

The cell invasion (Fig. 2a, b) and migration (Fig. 2c, d) abilities of glioma cells were dramatically decreased in the gefitinib-treated group, and were further inhibited in the gefitinib+miR-450a-5p mimics treated group (Fig. 2a–d). However, the gefitinib+miR-450a-5p inhibitor-treated group showed the increased invasion (Fig. S4a–b) and migration (Fig. S4c–d) abilities. As shown in Fig. 2e, f and Fig. S4e–f, for the EMT development, the expression of E-cadherin was significantly increased, while all other proteins were dramatically decreased after gefitinib treatment. Also, miR-450a-5p overexpression could further inhibit the EMT development, and miR-450a-5p downregulation was able to reverse the inhibition of EMT brought by gefitinib to a certain extent. All these results indicate that miR-450a-5p may synergize with gefitinib to inhibit the glioma cells migration, invasion, and EMT development. These conclusions were also confirmed with another EGFR inhibitor osimertinib (see Supplementary Results section and Supplemental Figs. S5–6 for further details).

### MiR-450a-5p regulates the EGFR signaling in glioma cells

**Fig. 3:**
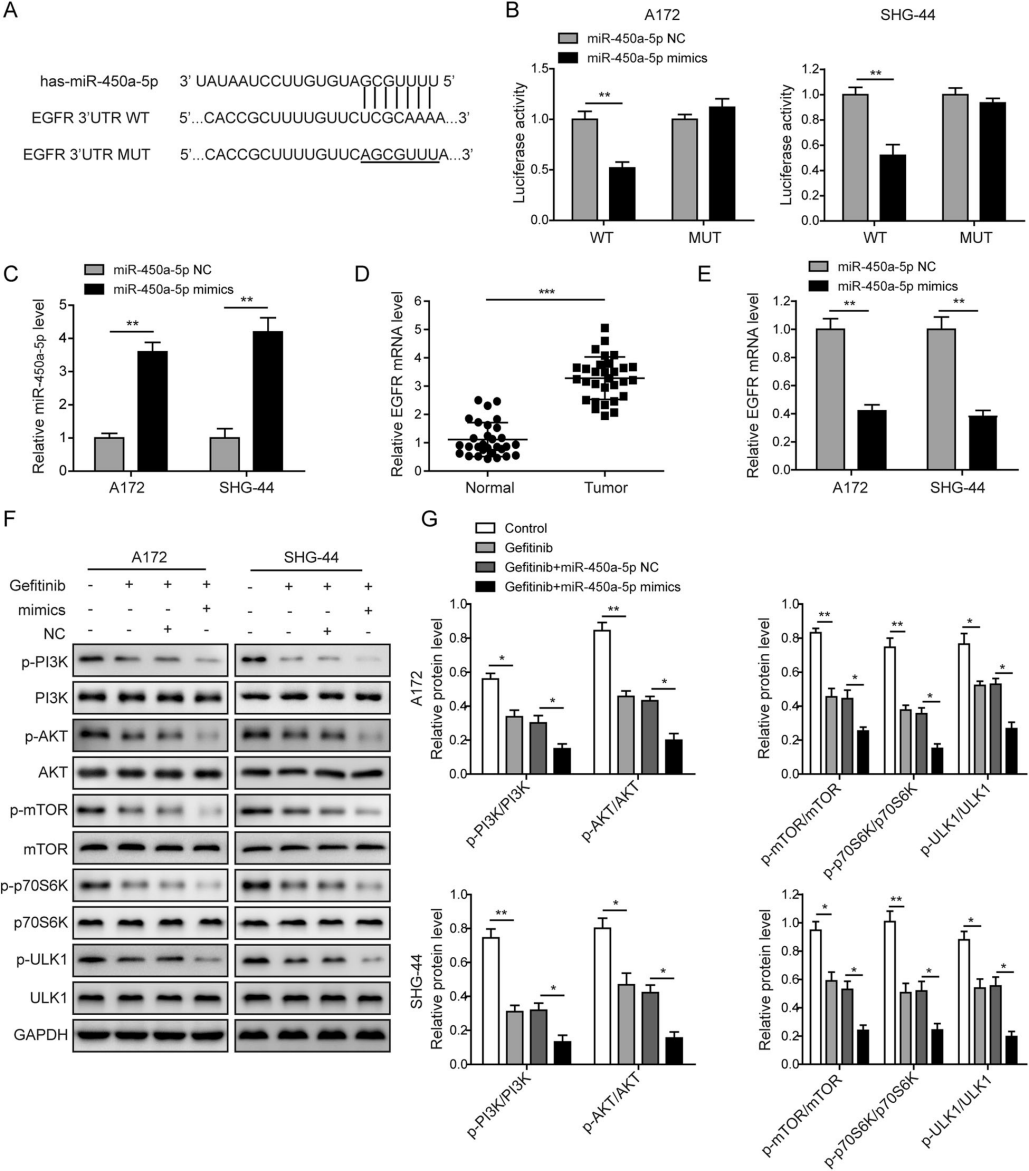
MiR-450a-5p regulates the EGFR signaling in glioma cells. **a** The binding sites between miR-450a-5p and EGFR WT and MUT 3′UTR. **b** The luciferase activities in the miR-450a-5p mimics or miR-450a-5p NC transfected glioma cells 48 h post-transfection. **c** The expression of miR-450a-5p in glioma cells detected by qRT-PCR 72 h post-transfection. **d** The expressions of EGFR in 30 pairs of glioma samples and corresponding normal tissues detected by qRT-PCR. **e** The expression of EGFR in glioma cells detected by qRT-PCR 72 h post-transfection. **f** Expression levels of the PI3K/AKT/mTOR signaling pathway related proteins detected by Western blot analysis. **g** Quantitative analysis of the Western blot results. In Western blotting, the transfected cells were cultured with DMEM medium containing gefitinib (20 μM for A172 cells and 40 μM for SHG-44 cells) for 48 h. The result was a representative of three independent experiments. Note: Error bars represented mean ± SD. *p* values were determined by one-way analysis of variance (ANOVA) followed by Tukey post hoc test. ***p < 0.001, **p < 0.01 and *p < 0.05.

The binding sites between miR-450a-5p and EGFR were indicated in Fig. 3a. As shown in Fig. 3b, the luciferase activity was significantly down-regulated when the miR-450a-5p was overexpressed in the wild type (WT) group, while no effect was observed in the mutant (MUT) group. Also, the expression of miR-450a-5p was dramatically increased, but the expression of EGFR was down-regulated after the miR-450a-5p overexpression (Fig. 3c, e). Furthermore, as shown in Fig. 3d, the expression of EGFR in the tumor tissues was significantly higher than that in the corresponding normal tissues. Also, clinicopathological characteristics of patients (Table S1) indicated that the EGFR expression was significantly correlated with the smoking status and WHO grades of the patients (*P* < 0.05), increased dramatically in tumor stages of III–IV. EGFR variant III (EGFRvIII) is the most common EGFR mutation that was reported in up to 30% of high-grade gliomas [25]. Our results showed that EGFRvIII was detected in about 50% of the glioma samples of stages of I–II, and which was detected in about 70% of the glioma samples of stages of III–IV (Fig. S7a–b). Moreover, as shown in Fig. 3f, g, the levels of phosphorylated-PI3K (p-PI3K), -AKT (p-AKT), -mTOR (p-mTOR), -p70S6K (p-p70S6K), and -ULK1 (p-ULK1) were significantly inhibited by gefitinib, which were further inhibited by the combined treatment of gefitinib and miR-450a-5p mimics. All these results indicate that miR-450a-5p exhibits a negative correlation with the EGFR, and regulates the EGFR-induced PI3K/AKT/mTOR signaling pathway in glioma cells.

### Overexpression of miR-450a-5p strengthens autophagy in glioma cells

**Fig. 4:**
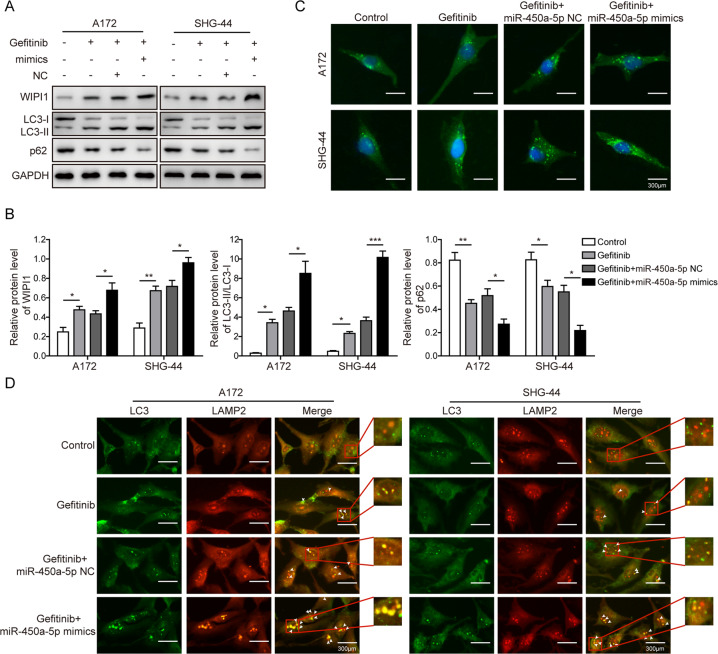
Overexpression of miR-450a-5p strengthens autophagy in glioma cells. **a** The expressions of autophagy-related proteins determined by Western blot analysis. **b** Quantitative analysis of the Western blot results. **c** The autophagosomes in both cell lines visualized by labeling the LC3. **d** The colocalization of LC3 and LAMP2 by immunofluorescence study. In all assays, the transfected cells were cultured with DMEM medium containing gefitinib (20 μM for A172 cells and 40 μM for SHG-44 cells) for 48 h. The result was a representative of three independent experiments. Note: Error bars represented mean ± SD. *p* values were determined by one-way analysis of variance (ANOVA) followed by Tukey post hoc test. ***p < 0.001, **p < 0.01 and *p < 0.05.

We further studied that whether miR-450a-5p may impact the autophagy in glioma cells. Firstly, the expression of WIPI1 and ratio of LC3-II/LC3-I increased dramatically, while the expression of p62 was decreased after gefitinib treatment, which were further strengthened by miR-450a-5p overexpression (Fig. 4a, b), while were reversed by miR-450a-5p downregulation (Fig. S8a–b). To further confirm this result, the immunofluorescence results (Fig. 4c) showed that the amount of autophagosomes was increased by the gefitinib, which was further increased by the combined treatment of gefitinib and miR-450a-5p overexpression. Moreover, as shown in Fig. 4d, more colocalization of LC3 and LAMP2 was observed after gefitinib treatment. In addition, miR-450a-5p overexpression further improved the fusion effect brought by gefitinib treatment on glioma cells. All these results indicate that miR-450a-5p can strengthen the induction of autophagy caused by gefitinib in glioma cells.

### Role of WIPI1 in miR-450a-5p-induced cell apoptosis and tumorigenesis in glioma cells

**Fig. 5:**
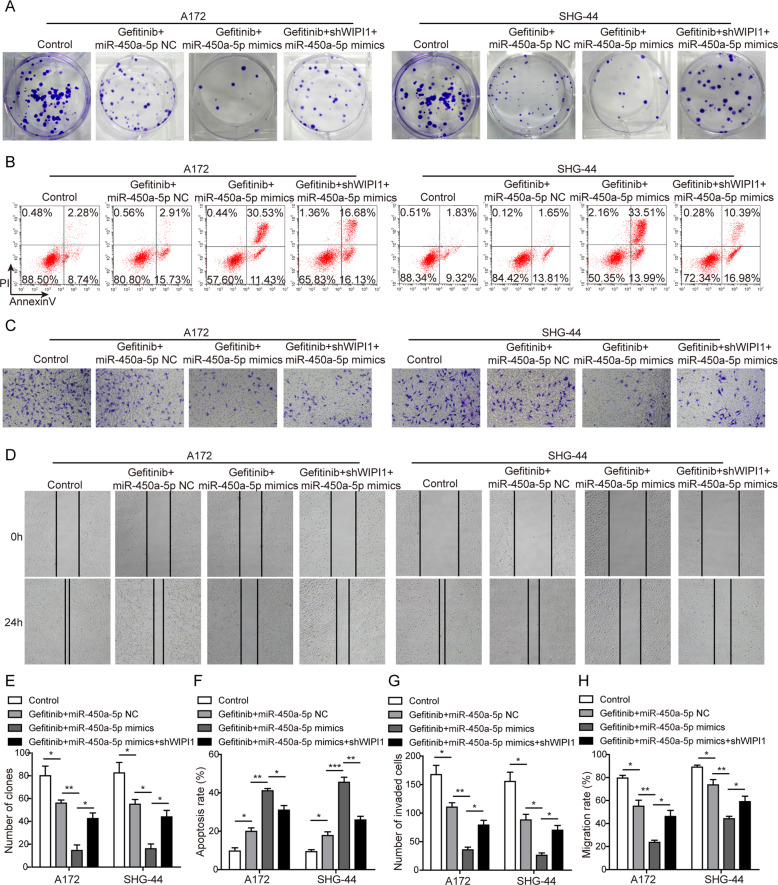
Role of WIPI1 in miR-450a-5p-induced cell apoptosis and tumorigenesis in glioma cells. **a** Effect of WIPI1 knockdown on the glioma cell proliferation tested by the colony formation assay. **b** Effect of WIPI1 knockdown on the glioma cell apoptosis detected by the flow cytometry. **c** Effect of WIPI1 knockdown on the invasion abilities of glioma cells detected by the Transwell invasion assay. **d** Effect of WIPI1 knockdown on the migration abilities of glioma cells detected by the wound healing assay. **e** Quantitative analysis of the numbers of colonies. **f** Quantitative analysis of apoptosis rate. **g** Quantitative analysis of the number of invaded cells. **h** Quantitative analysis of the migration rate. The transfected cells were treated with gefitinib (20 μM for A172 cells and 40 μM for SHG-44 cells). In the colony formation assay, the cells were cultured for 2 more weeks. In all other assays, the transfected cells were cultured for 48 h. The result was a representative of three independent experiments. Note: Error bars represented mean ± SD. *p* values were determined by one-way analysis of variance (ANOVA) followed by Tukey post hoc test. ***p < 0.001, **p < 0.01 and *p < 0.05.

Given the role of WIPI1 in autophagy, the function of WIPI1 in response to miR-450a-5p induction was studied. As shown in Fig. 5a, e, much fewer colonies were observed when the cells were treated with gefitinib and miR-450a-5p mimics, however, these effects were obviously reversed by the WIPI1 knockdown. Consistently, the apoptosis induced by gefitinib and miR-450a-5p was successfully reversed by WIPI1 knockdown (Fig. 5b, f). The cell invasion and migration abilities of glioma cells were significantly promoted by WIPI1 knockdown when compared with the gefitinib and miR-450a-5p mimics group (Fig. 5c, g; 5d, h). All these results indicate that WIPI1 knockdown can reverse the effects on proliferation, apoptosis, invasion, and migration induced by miR-450a-5p in glioma cells.

### Role of WIPI1 in miR-450a-5p-induced autophagy in glioma cells

**Fig. 6:**
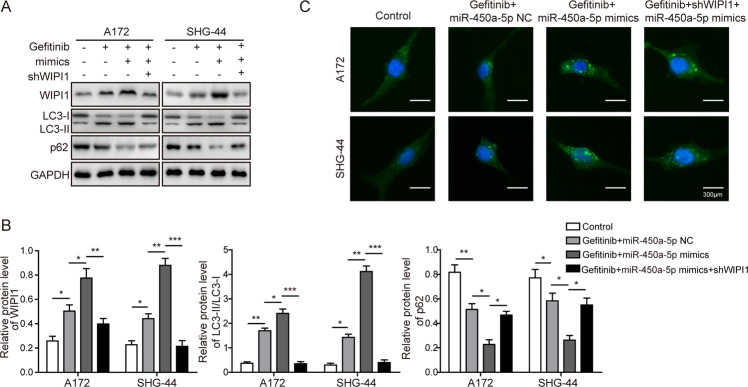
Role of WIPI1 in miR-450a-5p-induced autophagy in glioma cells. **a** The expression levels of autophagy-related proteins after WIPI1 knockdown determined by Western blot analysis. **b** Quantitative analysis of the Western blot results. **c** Effect of WIPI1 knockdown on the amount of autophagosomes determined by immunofluorescence. In all assays, the transfected cells were cultured with DMEM medium containing gefitinib (20 μM for A172 cells and 40 μM for SHG-44 cells) for 48 h. The result was a representative of three independent experiments. Note: Error bars represented mean ± SD. *p* values were determined by one-way analysis of variance (ANOVA) followed by Tukey post hoc test. ***p < 0.001, **p < 0.01 and *p < 0.05.

To further investigate the role of WIPI1 in miR-450a-5p-induced autophagy, the expression of WIPI1 and ratio of LC3-II/LC3-I were decreased dramatically when WIPI1 was knocked down compared with the gefitinib and miR-450a-5p treated group (Fig. 6a, b). As expected, the amount of autophagosomes was decreased obviously by WIPI1 knockdown (Fig. 6c). Therefore, we proposed that WIPI1 knockdown can reverse the effects induced by miR-450a-5p via blocking the progression of autophagy in glioma cells.

### Effects of miR-450a-5p overexpression on the glioma tumorigenesis in subcutaneous and intracranial models

**Fig. 7:**
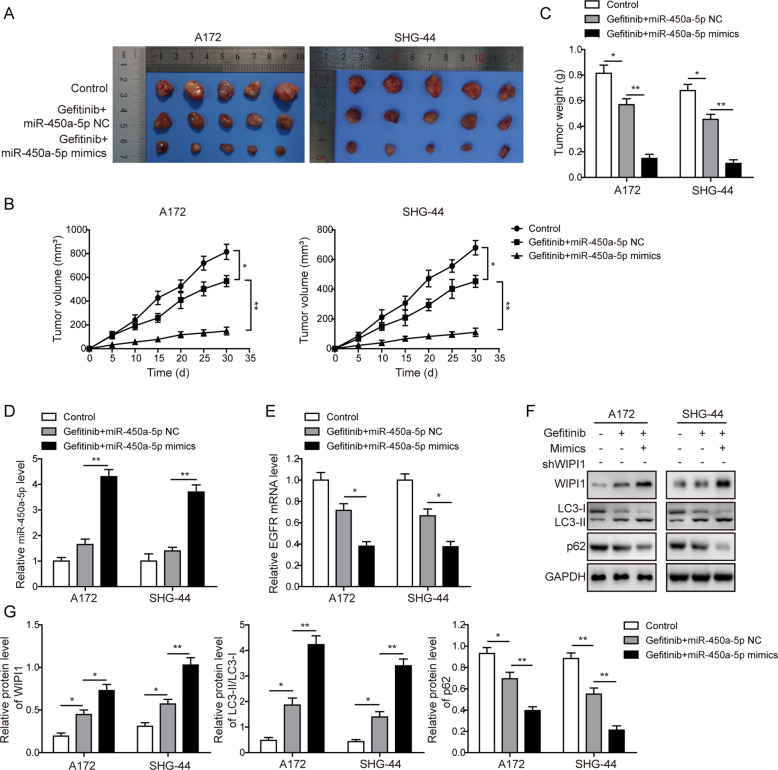
Effects of miR-450a-5p overexpression on the glioma tumorigenesis in subcutaneous model. **a** Tumor samples from differently treated mice. **b** Tumor volumes among different groups. **c** Tumor weights among different groups. **d** The expression level of miR-450a-5p among different tumor samples determined by qRT-PCR. **e** The expression level of EGFR among different tumor samples determined by qRT-PCR. **f** The expression levels of autophagy-related proteins among different tumor samples detected by Western blot analysis. **g** Quantitative analysis of the Western blot results. For subcutaneous xenograft model, 200 μL of cell suspension (about 1 × 10^7^) in PBS was subcutaneously injected into the nude mice. Tumor sizes were measured every 5 days with electronic caliper for 30 days. Tumor volume (V) was calculated by the formula: V = 0.5 × length × width^2^. The result was a representative of three independent experiments. Note: Error bars represented mean ± SD. *p* values were determined by one-way analysis of variance (ANOVA) followed by Tukey post hoc test. **p < 0.01 and *p < 0.05.

**Fig. 8:**
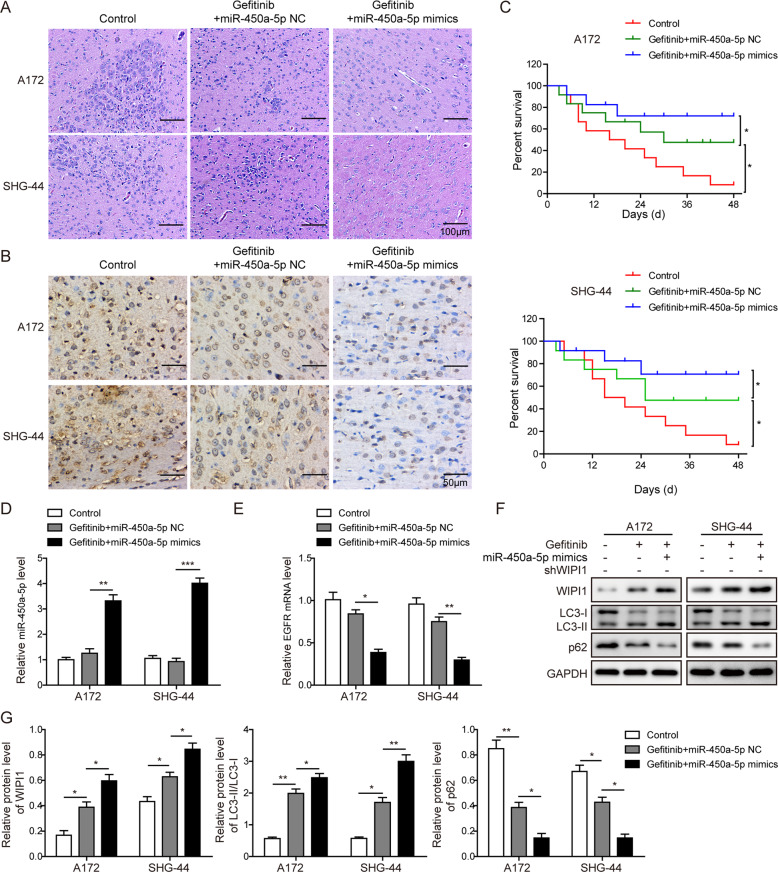
Effects of miR-450a-5p overexpression on the glioma tumorigenesis in intracranial model. **a** Representative images of HE staining from the brains of nude mice implanted intracranially with indicated glioma cells. Scale bar = 100 μm. **b** Representative images of immunohistochemical staining for Ki-67 expression in xenograft sections. Scale bar = 50 μm. **c** Kaplan–Meier survival analysis of overall survival. **d** The expression level of miR-450a-5p among different tumor samples determined by qRT-PCR. **e** The expression level of EGFR among different tumor samples determined by qRT-PCR. **f** The expression levels of autophagy-related proteins among different tumor samples detected by Western blot analysis. **g** Quantitative analysis of the Western blot results. For intracranial xenograft model, cells (about 1 × 10^6^) in 20 μL of serum-free DMEM were implanted intracranially. The result was a representative of three independent experiments. Note: Error bars represented mean ± SD. *p* values were determined by one-way analysis of variance (ANOVA) followed by Tukey post hoc test. ***p < 0.001, **p < 0.01 and *p < 0.05.

We next evaluated the influence of miR-450a-5p overexpression on the glioma tumorigenesis in vivo. In subcutaneous model, tumors from gefitinib+miR-450a-5p NC group were much smaller than those in the control group, and miR-450a-5p overexpression further decreased the tumor sizes (Fig. 7a, b). A same pattern was observed for the weights of tumors (Fig. 7c). Furthermore, we established orthotopic tumor model, the less glioma cell infiltration was observed in the site of implantation in gefitinib+miR-450a-5p NC group. MiR-450a-5p overexpression further decreased the amount of glioma cell infiltration (Fig. 8a). Level of Ki-67 was decreased in gefitinib+miR-450a-5p NC group, and miR-450a-5p overexpression further decreased the Ki-67 level (Fig. 8b). Overall survival of animals was also prolonged in gefitinib+miR-450a-5p NC group, and was further improved in gefitinib+miR-450a-5p mimics group (Fig. 8c). In both subcutaneous and intracranial models, the expression of miR-450a-5p was increased, while the expression of EGFR was down-regulated in the gefitinib + miR-450a-5p NC group, and these changes of miR-450a-5p and EGFR were further promoted by miR-450a-5p overexpression (Fig. 7d, e and Fig. 8d, e). Furthermore, the WIPI1 expression and the ratio of LC3-II/LC3-I were dramatically increased, while the expression of p62 was decreased in tumor samples of the gefitinib + miR-450a-5p NC group (Fig. 7f, g and Fig. 8f, g). Also, when combined the gefitinib treatment with miR-450a-5p overexpression, the effects on the expression of the autophagy-related proteins were further strengthened. These results further confirmed in vivo that miR-450a-5p could induce autophagy and further increase the gefitinib drug susceptibility of glioma cells.

## Discussion

At present, the treatment of glioma is mainly surgical resection combined with postoperative chemotherapy and radiotherapy [26]. More and more scholars have found that miRNA is closely related to EGFR-TKI sensitivity [27, 28]. MiR-16 and miR-136 increase the sensitivity of glioma cells to temozolomide by targeting Bcl-2 and AEG-1, respectively [29, 30]. MiR-21 down-regulates the activity of Wnt/β-catenin pathway and thus increases the tolerance of glioma to temozolomide [31]. In present study, we found that expression of miR-450a-5p was significantly lower, while the expression of EGFR was higher in the glioma tissue samples especially in metastatic glioma samples. MiR-450a-5p exhibited a negative correlation with the EGFR via targeting the 3′UTR of EGFR, and regulated the EGFR-induced PI3K/AKT/mTOR signaling pathway in glioma cells. As expected, the cell viability and proliferation were inhibited, while the cell apoptosis was promoted by gefitinib. In addition, miR-450a-5p was found to synergize with gefitinib to inhibit the glioma cells migration, invasion, and EMT development. Interestingly, all the effects brought by gefitinib were reversed by miR-450a-5p inhibition. MiR-450a-5p could also increase drug sensitivity of another EGFR inhibitor osimertinib in glioma. Therefore, miR-450a-5p may serve as a tumor suppressor gene in glioma. By regulating the expression of miR-450a-5p in glioma, it may improve the chemoresistance during glioma treatment. Also, combination treatment of miR-450a-5p and gefitinib also showed promotive apoptosis on normal glial cells. However, the apoptosis rates of normal glial cells induced by gefitinib alone or combination treatment were dramatically less than those in the glioma cells, which may be resulted from the lower expression of EGFR in normal cells than glioma cells. Although we cannot completely rule out this side effect on normal glial cells, we are able to reduce the dose of gefitinib, thereby reducing the side effects of chemotherapy drugs to patients through combining the use of miR-450a-5p and gefitinib. It was also well studied that p53 and IDH mutations in glioma were associated with gliomagenesis [32–35]. Therefore, we speculate that the mutation status of p53 and IDH may also affect the results of proliferation, apoptosis, migration, and invasion of glioma cells. The role of p53 and IDH status will be further explored in our future studies.

EGFR variant III (EGFRvIII) is the most common EGFR mutation that was reported in up to 30% of high-grade gliomas [25]. Our results also showed that EGFRvIII was detected in about 50% of the glioma samples of stages of I–II, and which was detected in about 70% of the glioma samples of stages of III–IV. We also have determined the EGFRvIII level in two glioma cells recruited in this study, but very low or no expression was observed (data not shown). Gefitinib is the first generation of EGFR inhibitors, and cells expressing EGFRvIII had increased resistance to gefitinib [36, 37]. Thus, it is worth studying the combination treatment of gefitinib and miR-450a-5p in glioma cells of EGFRvIII mutation in the future.

Intracellular mTOR has a higher phosphokinase activity, thereby inhibiting autophagy [38]. p70S6K is a known substrate protein of mTORC1, and its phosphorylation level indicates the activity of mTORC1. ULK (Unc-51-like kinase) is the homologous protein of Atg1, and mTORC1 can inhibit autophagy by phosphorylating ULK1. The p62/SQSTM1 protein acts to link the LC3 protein, and it is to be incorporated into autophagosomes. As the initial formation signal of autophagosomes, after the production of PI3P, WIPI1 protein can recruit downstream ATG molecules to initiate autophagy [20]. Therefore, p70S6K, ULK’s phosphorylation level, WIPI1, LC3-II/LC3-I, and p62/SQSTM1 are taken as the important indicators in this study for the detection of autophagy. Our results found that gefitinib treatment dramatically increased the expression of WIPI1 and ratio of LC3-II/LC3-I, and decreased the expression of p62. The autophagy was further induced via overexpressing miR-450a-5p. We also found that WIPI1 knockdown can reverse the miR-450a-5p-induced effects on the proliferation, apoptosis, invasion, and migration of glioma cells. We further confirmed that WIPI1 knockdown can negatively regulate the expressions of autophagy-related proteins and inhibit the formation of autophagosomes, which further indicated that miR-450a-5p can regulate the autophagy in glioma cells.

It is generally believed that there are three relationships between autophagy and apoptosis [39]: (a) autophagy is required for apoptosis: autophagy precedes apoptosis and upregulates the activity of the latter; (b) autophagy and apoptosis antagonize each other: autophagy can promote cell survival by inhibiting apoptosis; (c) autophagy and apoptosis synergistically promote cell death: the two can transform each other under certain conditions to form the “double insurance” of promoting cell death. In present study, miR-450a-5p was found to induce the cell apoptosis and promote the autophagy in glioma cells. As also reported by Li et al. [40], miR-519a was able to induce autophagy, and further enhance apoptosis in in vitro and in vivo glioma models. Therefore, in our case, we propose that the autophagy and apoptosis induced by miR-450a-5p synergistically promote cell death, which then increases the gefitinib chemosensitivity to glioma cells. It is also believed that the radiation treatment could cause protective autophagy allowing glioma cell survival [41]. With our current evidences, it’s still not clear how the combined treatment of gefitinib and miR-450a-5p would affect glioma cells post radiation. We speculate that this combination may partly counteract the protective effect induced by the radiation treatment in the anticancer therapy, and this relationship will be further investigated in our subsequent studies.

Meanwhile, in the case of miR-450a-5p overexpression, it may reduce the dose of gefitinib, thereby reducing the side effects of chemotherapy drugs to patients. Although targeted drugs are unlikely to kill all tumor cells, they can stop the tumors from growing or even make them shrink, especially playing an irreplaceable role in patients with ineffective radiotherapy and chemotherapy. On the basis of the important role of WIPI proteins in autophagy, our finding also demonstrated WIPI1 may also involve in the drug sensitivity of glioma cells to gefitinib. Hence, a better understanding of WIPI proteins could help to develop novel targets for rational therapies.

To sum up, we found that miR-450a-5p can cooperate with gefitinib to inhibit the glioma tumorigenesis through inducing autophagy by regulating EGFR-induced PI3K/AKT/mTOR signaling pathway in glioma cells, thereby enhancing the drug sensitivity of glioma cells to gefitinib.

## Materials and methods

See Supplementary Materials and Methods information for details.

## Supplementary information

Supplementary Figure Legends

Supplementary Materials and Methods

Supplementary Results

Supplemental figure S1

Supplemental figure S2

Supplemental figure S3

Supplemental figure S4

Supplemental Table S1

Supplemental figure S5

Supplemental figure S6

Supplemental figure S7

Supplemental figure S8
