# Supplementary material for: MiR-450a-5p strengthens the drug sensitivity of gefitinib in glioma chemotherapy via regulating autophagy by targeting EGFR
Source: Oncogene. 2020 Aug 20;39(39):6190–202. doi: 10.1038/s41388-020-01422-9 (PMC7515841; doi:10.1038/s41388-020-01422-9)
Supplement: Supplementary file 1 — Supplementary Figure Legends [file 41388_2020_1422_MOESM1_ESM.docx]

**Supplementary Information**

**Supplementary Figure Legends**

**Supplemental Figure S1. Inhibition of miR-450a-5p moderately reverses the impact of gefitinib on the glioma cells proliferation and apoptosis.** (A) The cell proliferation after different treatments determined by the colony formation assay. (B) Quantitative analysis of the numbers of colonies. (C) Effect of miR-450a-5p inhibition and gefitinib on the cell apoptosis investigated by flow cytometry. (D) Quantitative analysis of apoptosis rate. (E) Expression levels of cell apoptosis related proteins, including Bcl-2, Bax, cleaved caspase-3 and uncleaved PARP, detected by Western blot analysis. (F) Quantitative analysis of the Western blot results. In the colony formation assay, the transfected cells were treated with gefitinib (20 μM for A172 cells and 40 μM for SHG-44 cells) and cultured for 2 more weeks. In all other assays, the transfected cells were cultured with DMEM medium containing gefitinib (20 μM for A172 cells and 40 μM for SHG-44 cells) for 48 h. The result was a representative of three independent experiments. Note: Error bars represented mean ± SD. *p* values were determined by one-way analysis of variance (ANOVA) followed by Tukey post hoc test. ** *p*< 0.01 and * *p*< 0.05.

**Supplemental Figure S2. Effects of miR-450a-5p overexpression or inhibition on normal glial cells.** (A) The levels of miR-450a-5p in normal glial cells after transfection of miR-450a-5p mimics or inhibitor detected by qRT-PCR. (B) The levels of EGFR mRNA in normal glial cells after transfection of miR-450a-5p mimics or inhibitor detected by qRT-PCR. (C) The protein levels of EGFR in normal glial cells after transfection of miR-450a-5p mimics or inhibitor determined by Western blotting. (D) Quantitative analysis of the Western blot results. (E) Effect of miR-450a-5p on the cell apoptosis of normal glial cells investigated by flow cytometry. (F) Quantitative analysis of apoptosis rate. (G) Expression levels of cell apoptosis related proteins, including Bcl-2, Bax, cleaved caspase-3 and uncleaved PARP detected by Western blot analysis. (H) Quantitative analysis of the Western blot results. In all assays, the normal glial cells were transfected with the miR-450a-5p mimics or inhibitor for 48 h, then performed the above experiments. The result was a representative of three independent experiments. Note: Error bars represented mean ± SD. *p* values were determined by one-way analysis of variance (ANOVA) followed by Tukey post hoc test. *** *p*< 0.001, ** *p*< 0.01 and * *p*< 0.05.

**Supplemental Figure S3. MiR-450a-5p synergizes with gefitinib to impact the normal glial cells apoptosis.** (A) Effect of miR-450-5p overexpression and gefitinib on the cell apoptosis of normal glial cells investigated by flow cytometry. (B) Quantitative analysis of apoptosis rate. (C) Expression levels of cell apoptosis related proteins, including Bcl-2, Bax, cleaved caspase-3 and uncleaved PARP detected by Western blot analysis. (D) Quantitative analysis of the Western blot results. In all assays, the transfected cells were cultured with DMEM medium containing gefitinib (40 μM) for 48 h. The result was a representative of three independent experiments. Note: Error bars represented mean ± SD. *p* values were determined by one-way analysis of variance (ANOVA) followed by Tukey post hoc test. *** *p*< 0.001, ** *p*< 0.01 and * *p*< 0.05.

**Supplemental Figure S4. Inhibition of miR-450a-5p moderately reverses the impact of gefitinib on the glioma cells migration and invasion**. (A) The cell invasion abilities of glioma cells detected by the Transwell invasion assay. (B) Quantitative analysis of the invaded cells. (C) The cell migration abilities of glioma cells detected by the wound healing assay. (D) Quantitative analysis of the migration rate. (E) Expression levels of EMT related proteins, including Fibronectin, MMP-2, MMP-9, E-cadherin, N-cadherin, and Vimentin detected by Western blot analysis. (F) Quantitative analysis of the Western blot results. In all assays, the transfected cells were cultured with DMEM medium containing gefitinib (20 μM for A172 cells and 40 μM for SHG-44 cells) for 48 h. The result was a representative of three independent experiments. Note: Error bars represented mean ± SD. *p* values were determined by one-way analysis of variance (ANOVA) followed by Tukey post hoc test. ** *p*< 0.01 and * *p*< 0.05.

**Supplemental Figure S5. MiR-450a-5p synergizes with osimertinib to impact the glioma cells proliferation and apoptosis.** (A) Effect of miR-450a-5p and osimertinib on the cell proliferation of A172 and SHG-44 cells detected by the MTT assay. (B) The cell proliferation after different treatments determined by the colony formation assay. (C) Quantitative analysis of the numbers of colonies. (D) Effect of miR-450a-5p and osimertinib on the cell apoptosis investigated by flow cytometry. (E) Quantitative analysis of apoptosis rate. (F) Expression levels of cell apoptosis related proteins, including Bcl-2, Bax, cleaved caspase-3 and uncleaved PARP, detected by Western blot analysis. (G) Quantitative analysis of the Western blot results. In the colony formation assay, the transfected cells were treated with osimertinib (2.5 μM) and cultured for 2 more weeks. In all other assays, the transfected cells were cultured with DMEM medium containing osimertinib (2.5 μM) for 48 h. The result was a representative of three independent experiments. Note: Error bars represented mean ± SD. *p* values were determined by one-way analysis of variance (ANOVA) followed by Tukey post hoc test. *** *p*< 0.001, ** *p*< 0.01 and * *p*< 0.05.

**Supplemental Figure S6. MiR-450a-5p synergizes with osimertinib to impact the glioma cells migration and invasion**. (A) The cell invasion abilities of glioma cells detected by the Transwell invasion assay. (B) Quantitative analysis of the invaded cells. (C) The cell migration abilities of glioma cells detected by the wound healing assay. (D) Quantitative analysis of the migration rate. (E) Expression levels of EMT related proteins, including Fibronectin, MMP-2, E-cadherin and N-cadherin detected by Western blot analysis. (F) Quantitative analysis of the Western blot results. In all assays, the transfected cells were cultured with DMEM medium containing osimertinib (2.5 μM) for 48 h. The result was a representative of three independent experiments. Note: Error bars represented mean ± SD. *p* values were determined by one-way analysis of variance (ANOVA) followed by Tukey post hoc test. *** *p*< 0.001,** *p*< 0.01 and * *p*< 0.05.

**Supplemental Figure S7. The expression levels of EGFRvIII in glioma samples**. (A) The expression of EGFRvIII in 30 glioma samples (13 cases of glioma samples in tumor stages of I-II and 17 cases of glioma samples in tumor stages of III-IV) detected by Western blotting. (B) Ratio of EGFRvIII positive samples in tumor stages of I-II and III-IV. The result was a representative of three independent experiments. Note: Error bars represented mean ± SD. *p* values were determined by Student’s *t* test. * *p*< 0.05.

**Supplemental Figure S8. Inhibition of miR-450a-5p moderately reverses the impact of gefitinib on the glioma cells autophagy**. (A) The expressions of autophagy related proteins (WIPI1, LC3-I, LC3-II, and p62) determined by Western blot analysis. (B) Quantitative analysis of the Western blot results. In Western blotting, the transfected cells were cultured with DMEM medium containing gefitinib (20 μM for A172 cells and 40 μM for SHG-44 cells) for 48 h. The result was a representative of three independent experiments. Note: Error bars represented mean ± SD. *p* values were determined by one-way analysis of variance (ANOVA) followed by Tukey post hoc test. *** *p*< 0.001, ** *p*< 0.01 and * *p*< 0.05.
