# Supplementary material for: MiR-450a-5p strengthens the drug sensitivity of gefitinib in glioma chemotherapy via regulating autophagy by targeting EGFR
Source: Oncogene. 2020 Aug 20;39(39):6190–202. doi: 10.1038/s41388-020-01422-9 (PMC7515841; doi:10.1038/s41388-020-01422-9)
Supplement: Supplementary file 2 — Supplementary Materials and Methods [file 41388_2020_1422_MOESM2_ESM.docx]

**Supplementary Information**

**Materials and Methods**

**Clinical specimens**

A total of 30 patients pathologically diagnosed with glioma in the third Xiangya Hospital, Central South University (Changsha, China) were recruited in this study. None of the patients received either radiotherapy or chemotherapy prior to surgery. The clinicopathological characteristics of these patients were listed in **Table S1**. The performance status of the subjects was scored according to the Karnofsky Performance Scale (KPS) scoring system as previously described [1], with a score range between 0 and 100. Pathological stage of the glioma was performed according to the tumor grading system outlined by the World Health Organization (WHO). All tissue samples were stored at −80°C until use. The present study was approved by the Ethics Committee of the third Xiangya Hospital and all patients provided written informed consent.

**Cell culture and treatment**

The human glioma cell lines A172 and SHG-44, and normal human astrocytes (NHA) were purchased from the American Type Culture Collection (ATCC, Manassas, VA, USA) or Lonza (Walkersville, MD, USA). All the cell lines included in this study have been authenticated by STR profiling and tested for mycoplasma contamination. Cells were cultured in Dulbecco’s Modified Eagle’s Medium (DMEM, Sigma‐Aldrich, USA) supplemented with 10% fetal bovine serum (FBS), 2 mM L-glutamine, and 100 U/ml penicillin-streptomycin, and incubated at 37°C and 5% CO_2_. For gefitinib or osimertinib treatment, 200 mM gefitinib (Astra Zeneca, UK) or 100 mM osimertinib (Astra Zeneca, UK) stock solution in dimethyl sulfoxide (DMSO) was prepared for the following experiments.

**Cell transfections**

The overexpression or inhibition of miR-450a-5p was carried out by transfection with the miR-450a-5p mimics or inhibitor (GenePharma, Shanghai, China). The cells were plated in 6-well plates for 24 h and then transfected for 24 h with the miR-450a-5p mimics or inhibitor using Lipofectamine 2000 (Thermo Fisher Scientific, USA). The short hairpin RNA (shRNA) targeting WIPI1, and scrambled control shRNA were obtained from GenePharma (China). Cells were cultured in 6-well plates, respectively, and were transfected with shWIPI1 or shNC for 24 h according to the manufacturer’s introduction, using RNAi-Mate transfection reagent (GenePharma, China).

**Stable cell line generation for *in vivo* study**

MiR-450a-5p expressing lentivirus was generated by GenePharma (China). Virus production and cell transduction in glioma cells were performed as previously described [2]. A172 and SHG-44 cells (2.5 × 10^5^ cells/well) were seeded in 24-well plates. In the following day, the cells were transducted with miR-450a-5p-expressing or NC lentiviruses and medium was replaced 16 h after adding viruses. Transduced cells were further expanded to generate low-passage stocks of stable cell lines. These stable cell lines were used for the following *in vivo* studies.

**MTT assay**

Cell proliferation of glioma cells was detected using MTT assay (Sigma, USA). Cells were divided into three groups: gefitinib treated group, gefitinib+miR-450a-5p NC treated group, gefitinib+miR-450a-5p mimics treated group. The transfected cells were cultured into 96-well plates with 100 μL of DMEM medium containing various concentrations of gefitinib (0, 2.5, 5, 10, 20, 40, 80 μM). After culturing for 48 h, the medium was replaced with fresh culture medium containing 10 μL of 5 mg/mL MTT reagent. After 4 h incubation at 37°C, the MTT solution was replaced with 150 μL of DMSO. The absorbance at 490 nm was then detected with a microplate reader (BioTek, USA). In some cases, the cells were also divided into three groups: osimertinib treated group, osimertinib+miR-450a-5p NC treated group, osimertinib+miR-450a-5p mimics treated group. The transfected cells were treated with various concentrations of osimertinib (0, 0.625, 1.25, 2.5, 5, 10, 20 μM).

**Flow cytometry analysis of cell apoptosis**

Apoptotic cells were identified using the Annexin V-FITC Apoptosis Detection Kit (Sigma-Aldrich, USA) according to the manufacturer’s instructions. The transfected cells were cultured into 24-well plates with 1 mL of DMEM medium containing gefitinib (20 μM for A172 cells and 40 μM for SHG-44 cells and normal human astrocytes) or osimertinib (2.5 μM). After culturing for 48 h, the cells were collected, washed twice with cold PBS and resuspended in 1× binding buffer. The cells were stained with 5 μL Annexin V-FITC for 15 min and then 5 μl PI for 10 min in the dark at room temperature. The cells were examined using a FACSCanto II flow cytometer (BD Biosciences, Germany).

**Colony formation assay**

The transfected cells were cultured for 24 h and 2×10^2^/well transfected cells were plated in 6-well plates with gefitinib (20 μM for A172 cells and 40 μM for SHG-44 cells) or osimertinib (2.5 μM) and cultured for 2 more weeks. The plates were then washed with PBS for twice, and cells were fixed with methanol-acetic acid. At last, the cells were stained with 0.5% cristal violet. The number of colonies was counted under a microscope (Olympus, Japan). The counting process was conducted by an assessor blind to treatment allocation.

**Wound healing assay**

The transfected cells (2.5×10^5^/well) were cultured into 24-well plates with 1 mL of DMEM medium containing gefitinib (20 μM for A172 cells and 40 μM for SHG-44 cells and normal human astrocytes) or osimertinib (2.5 μM). After culturing for 24 h at 37°C, the confluent transfected cells were scratched with a 200 µL pipette tip. After 24 h incubation, plates were washed with fresh medium to remove non-adherent cells and then photographed. Wound area was determined using an inverted microscope (IX71; Olympus, Japan). Distances were measured and analyzed by ImageJ software (NIH, US). The migration distances of cells were measured by the following formula: (W_0h_–W_24h_)/W_0h_×100%. The measuring process was conducted by an assessor blind to treatment allocation.

**Cell invasion assay**

Cell invasion assay was performed using a Transwell system (Corning, USA). The upper chamber of the Transwell was coated with extracellular matrix (BD Biosciences, USA). The transfected cells were cultured into 24-well plates with 1 mL of DMEM medium containing gefitinib (20 μM for A172 cells and 40 μM for SHG-44 cells) or osimertinib (2.5 μM). After culturing for 48 h, cells were trypsinized, and 1×10^5^ cells were seeded into the upper chamber with serum free opti-MEM medium. The low chamber was filled with 800 µL medium containing 10% FBS as a chemoattractant. After incubation for 48 h, the non-invading cells on the upper surface were erased with a cotton swab and cells on the lower side of the filter were fixed in 3.8% formaldehyde for 20 min and stained with 0.1% crystal violet solution. The number of cells in five randomly selected fields was counted under a light microscope (Olympus, Japan) and analyzed statistically. The accessing process was conducted by an assessor blind to treatment allocation.

**Luciferase reporter assay**

The EGFR 3’untranslated region (UTR) clones including wild type (WT) 3’UTR and mutant (MUT) 3’UTR were designed and synthesized by Sangon Biotech (Shanghai, China). EGFR 3’UTR was individually cloned into pGL4 luciferase reporter plasmid (Promega, USA). Then, cells were co-transfected with pGL4-EGFR or control reporter pGL4 reporter plasmids and miR-450a-5p mimics using Lipofectamine 2000 Reagent (Invitrogen). After culturing for 48 h, the relative luciferase activity was measured using Dual-Glow Luciferase Assay kit (Promega, USA) and a GloMax-Multi Detection System (Promega, USA).

**Immunofluorescence assay**

The transfected cells were cultured on glass coverslips with 1 mL of DMEM medium containing gefitinib (20 μM for A172 cells and 40 μM for SHG-44 cells) for 48 h and then fixed in 4% paraformaldehyde in PBS for 10 min on ice, permeabilized using 0.3% Triton-X100/PBS, and subsequently blocked with 4% BSA in PBS (Roche, Netherlands). Cells were incubated with rabbit anti-human LC-3 (#12741, Cell Signaling Technology, USA) and LAMP2 (ab25631, Abcam, USA) antibodies (1:1000 dilution) in blocking buffer for 2 h at room temperature, and washed three times with TBST (Tris-buffered saline, 0.1% Tween-20). Coverslips were then incubated with goat anti-rabbit fluorophore-conjugated secondary antibody (1:500; Invitrogen, US) in blocking buffer for 1 h at room temperature. Coverslips were washed again with TBST three times for 5 min each, and nucleus was stained with DAPI (1:1000; Santa Cruz, USA) for 5 min at room temperature. Finally, coverslips were mounted on glass slides with mounting media (DAKO) and analyzed by using the Zeiss LSM 700 confocal laser microscope.

**Nude mice subcutaneous and intracranial xenograft model**

The animal experiments were approved by the Guidelines for the Care and Use of Laboratory Animals and the Medical Ethics Committee of the third Xiangya Hospital (Changsha, China). 90 female six-week-old male BALB/c nude (nu/nu) mice were purchased from SJA Laboratory Animal Company (Hunan, China). The mice were randomly divided into three groups: 1) untreated glioma cells (control group); 2) glioma cells treated with gefitinib and miR-450a-5p NC group; 3) glioma cells treated with gefitinib and miR-450a-5p mimics group. For subcutaneous xenograft model, 200 μL of cell suspension (about 1×10^7^) in PBS was subcutaneously injected into the nude mice. Tumor sizes were measured every 5 days with electronic caliper for 30 days. Tumor volume (V) was calculated by the formula: V= 0.5×length×width^2^. All mice were sacrificed by cervical dislocation at day 30 after treatments. Tumor samples were collected and weighed for all groups. For intracranial xenograft model, cells (about 1×10^6^) in 20 μL of serum‐free DMEM were implanted intracranially as previous reported [3]. Overall survival was assessed by Kaplan-Meier analysis for about 50 days. Excised tumor tissues were fixed, paraffin-embedded, and sectioned. Obtained sections were then underwent haematoxylin and eosin (HE) staining and immunohistochemistry analysis for Ki-67 (#12202, Cell Signaling Technology, USA). The accessing process was conducted by an assessor blind to treatment allocation.

**Western blot analysis**

The transfected cells were cultured into 24-well plates with 1 mL of DMEM medium containing gefitinib (20 μM for A172 cells and 40 μM for SHG-44 cells) or osimertinib (2.5 μM). Cells were harvested after culturing for 48 h. Glioma samples and the harvested cells were lysed in the RIPA buffer (Sigma-Aldrich, USA). Protein concentrations were determined using the BCA protein assay kit (Thermo Fisher Scientific, USA). Proteins (30 μg) were separated by 10% SDS-PAGE and transferred onto a nitrocellulose membrane. After blocking with BSA, the membranes were then incubated with primary antibodies all from Cell Signaling Technology unless indicated otherwise: EGFR (#4267) and EGFRvIII (#250632, Abbiotec, San Diego, CA, USA), cell apoptosis related proteins p53 (#2527), Bcl-2 (#4223), Bax (#5023), cleaved caspase-8 (#9496), cleaved caspase-9 (#20750), cleaved caspase-3 (#9664), uncleaved PARP (#9542), and cleaved PARP (#5625), EMT related proteins fibronectin (#26836), MMP-2 (#40994), MMP-9 (#13667), E-cadherin (#3195), N-cadherin (#13116), and vimentin (#5741), PI3K/AKT/mTOR signaling pathway related proteins PI3K (#4263), p-PI3K (#13857), AKT (#4691), p-AKT (#4060), mTOR (#2893), p-mTOR (#5536), p70S6K (#2708), p-p70S6K (#97596), ULK1 (#8054), and p-ULK1 (#5869), autophagy related proteins WIPI1 (#12124), LC3 (#4108), and p62 (#8025) and GAPDH (#5174). GAPDH was loaded as an internal reference. Bands were then treated with the goat anti-rabbit IgG-HRP secondary antibody (ab6721, Abcam, USA). Bands were developed using chemiluminescence substance (Thermo Scientific, USA). The proteins were quantified using Quantity One software (Bio-Rad Laboratories, Inc., Hercules, CA, USA).

**RNA extraction and qRT-PCR**

Total RNA was isolated from glioma tissue samples and cells with TRIzol reagent (Invitrogen, USA) and RNeasy Plus Micro Kit (QIAGEN, USA) according to the manufacturer’s instructions. Then, reverse transcription was conducted to synthesize the cDNA by utilizing the SuperScript® IV First-Strand Synthesis System (Invitrogen, USA). qRT-PCR was performed in Applied Biosystems 7500 Real Time PCR System (Applied Biosystems, USA), using 20 ng template in 25 µL reaction volume with 2×Power SYBR® Green PCR Master Mix (Invitrogen, USA) and gene specific primer pairs for miRNA-450a-5p, EGFR, U6, and GAPDH in glioma cells. Amplification conditions were as follows: 95°C for 10 min followed by 45 cycles consisting of 95°C for 15 s, 58 °C for 30 s and 68°C for 60 s. The gene expression levels for all samples were normalized to U6 (for miRNA) or GAPDH expression using the 2^−ΔΔCt^ method.

**Statistical analysis**

Data were analyzed with Prism 6.0 (GraphPad Software, USA). All the data meet the assumption of normal distribution. All experiments were performed in at least three biological replicates, and each biological replicate contained three technical replicates. All data were expressed as the mean ± standard deviation (SD). Statistical evaluation was performed using Student’s *t* test (two tailed) between two groups or one-way analysis of variance (ANOVA) followed by Tukey post hoc test for multiple comparison. The correlation between miR-450a-5p/EGFR expression and clinicopathological characteristics of patients with glioma was assessed by the Chi-squared test. *P* < 0.05 was considered significantly different.

**References**

1 Peus D, Newcomb N, Hofer S. Appraisal of the Karnofsky Performance Status and proposal of a simple algorithmic system for its evaluation. *BMC Med Inform Decis Mak* 2013; 13: 72.

2 Frenster JD, Inocencio J, Placantonakis DG. Lentiviral Transduction of Primary Human Glioblastoma Cultures. *Methods Mol Biol* 2018; 1741: 81-89.

3 Feng Z, Zhou W, Wang J, Qi Q, Han M, Kong Y *et al*. Reduced expression of proteolipid protein 2 increases ER stress-induced apoptosis and autophagy in glioblastoma. *J Cell Mol Med* 2019.
