# Supplementary material for: MiR-450a-5p strengthens the drug sensitivity of gefitinib in glioma chemotherapy via regulating autophagy by targeting EGFR
Source: Oncogene. 2020 Aug 20;39(39):6190–202. doi: 10.1038/s41388-020-01422-9 (PMC7515841; doi:10.1038/s41388-020-01422-9)
Supplement: Supplementary file 3 — Supplementary Results [file 41388_2020_1422_MOESM3_ESM.docx]

**Supplementary Information**

**Results**

**Effects of miR-450a-5p overexpression or inhibition on normal glial cells.**

Transfections with miR-450a-5p mimics and inhibitor were able to regulate the levels of miR-450a-5p in normal glial cells successfully (Fig. S2A). As shown in Fig. S2B-D, the mRNA and protein levels of EGFR were negatively regulated by miR-450a-5p. MiR-450a-5p overexpression significantly promoted the cell apoptosis while downregulation of miR-450a-5p exerted the inverse effect on the apoptosis of normal glial cells (Fig. S2E-F). A consistent pattern was also observed by analyzing Bcl-2, Bax, cleaved caspase-3 and uncleaved PARP (Fig. S2G-H).

**MiR-450a-5p synergizes with gefitinib to impact the normal glial cells apoptosis.**

In addition, the effects of combination treatment of miR-450-5p overexpression and gefitinib (40 μM) on normal glial cell apoptosis were also determined. As shown in Fig. S3A-B, gefitinib could also promote the cell apoptosis of the normal glial cells. This effect was further strengthened by miR-450-5p mimics. This conclusion was confirmed by the protein levels of Bcl-2, Bax, cleaved caspase-3 and uncleaved PARP (Fig. S3C-D). In summary, combination treatment of miR-450-5p overexpression and gefitinib also showed promotive effects on apoptosis of normal glial cells.

**MiR-450a-5p is also able to increase drug sensitivity of osimertinib in glioma**

To further confirm the role of miR-450a-5p in the drug sensitivity of EGFR inhibitor, another EGFR inhibitor osimertinib was recruited in this study. Osimertinib is a third-generation EGFR-TKI which has been used to treat non-small-cell lung carcinomas with a specific mutation [1]. Our results showed that the cell viability of both A172 and SHG-44 cells was decreased after osimertinib treatment in a concentration-dependent manner (Fig. S5A). Also, overexpression of miR-450a-5p might also enhance the drug sensitivity of osimertinib to glioma cells. As the same as the gefitinib treated experiments, the proliferation and apoptosis of glioma cells after the treatment of osimertinib with or without miR-450a-5p overexpression were investigated. Much fewer colonies were observed when the cells were treated with osimertinib or transfected with miR-450a-5p mimics (Fig. S5B-C). Consistently, the cell proliferation was further inhibited in the miR-450a-5p overexpressed and osimertinib treated cells (Fig. S5B-C). Moreover, osimertinib treatment or miR-450a-5p mimics transfection promoted the cell apoptosis of both A172 and SHG-44 cells, respectively, which was further induced after miR-450a-5p overexpression in osimertinib treated cells (Fig. S5D-E). The Western blotting results further confirmed the cell apoptosis (Fig. S5F-G). All these results indicate that miR-450a-5p may also synergize with osimertinib to inhibit the glioma cell proliferation and induce the cell apoptosis.

The cell migration, invasion as well as the EMT development were further investigated after osimertinib treatment. The Transwell invasion assay showed that the cell invasion abilities of glioma cells were dramatically decreased in the osimertinib treated group, and were further inhibited by miR-450a-5p mimics (Fig. S6A-B). Consistently, the wound healing assay showed that miR-450a-5p overexpression can further enhance the inhibition of migration of glioma cells induced by osimertinib treatment (Fig. S6C-D). The expression level of E-cadherin was significantly increased and all other proteins were dramatically decreased in the osimertinib treated cells, and the above effects were reinforced by miR-450a-5p mimics (Fig. S6E-F). All these results indicate that miR-450a-5p may also synergize with osimertinib to inhibit the glioma cell migration, invasion, and EMT development.

**References**

1 Takeda M, Nakagawa K. First- and Second-Generation EGFR-TKIs Are All Replaced to Osimertinib in Chemo-Naive EGFR Mutation-Positive Non-Small Cell Lung Cancer? *Int J Mol Sci* 2019; 20.
