# Supplementary figures and images for: MiR-450a-5p strengthens the drug sensitivity of gefitinib in glioma chemotherapy via regulating autophagy by targeting EGFR
Source: Oncogene. 2020 Aug 20;39(39):6190–202. doi: 10.1038/s41388-020-01422-9 (PMC7515841; doi:10.1038/s41388-020-01422-9)

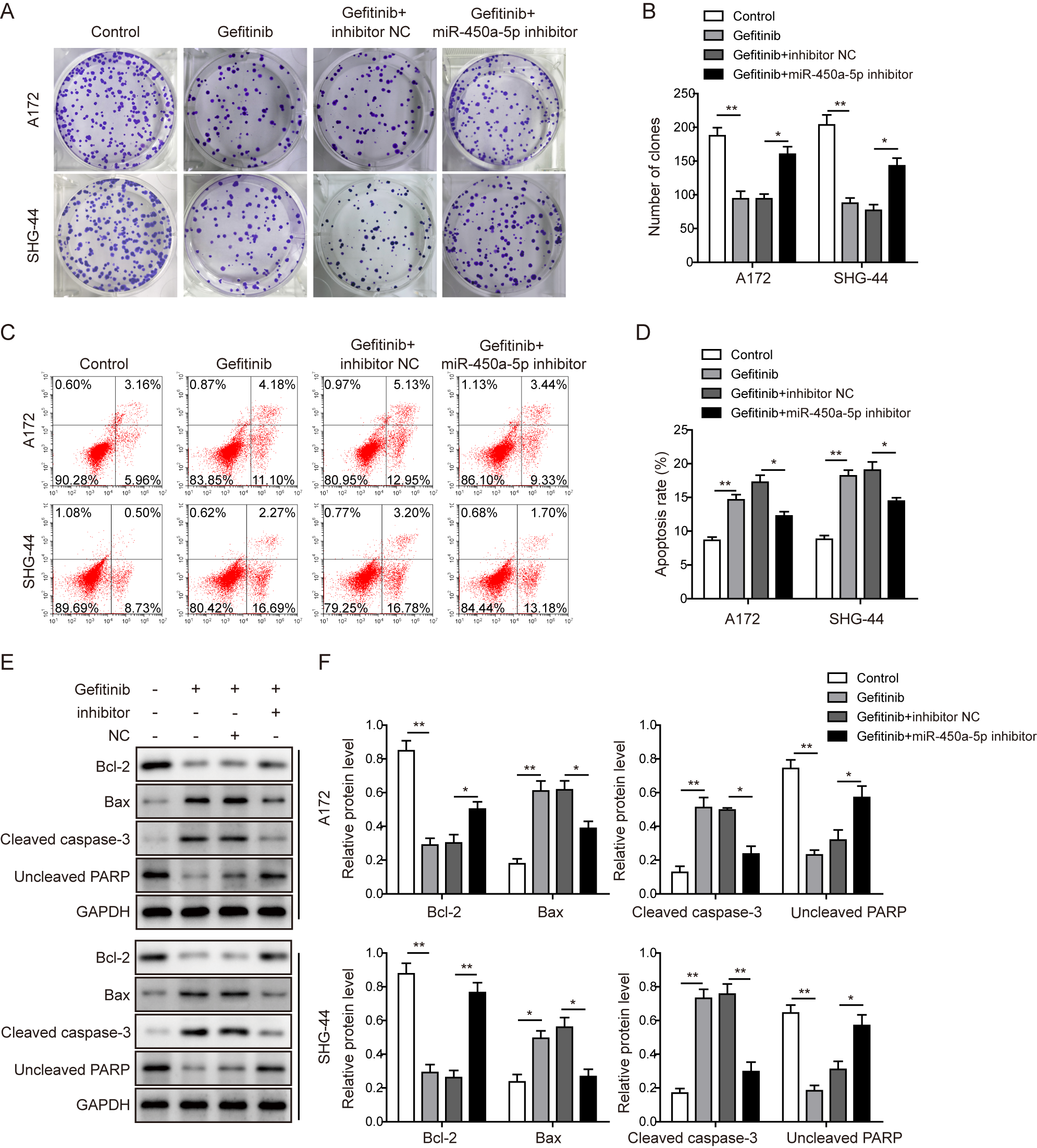

Supplement: Supplementary file 4 — Supplemental figure S1 [file 41388_2020_1422_MOESM4_ESM.tif]

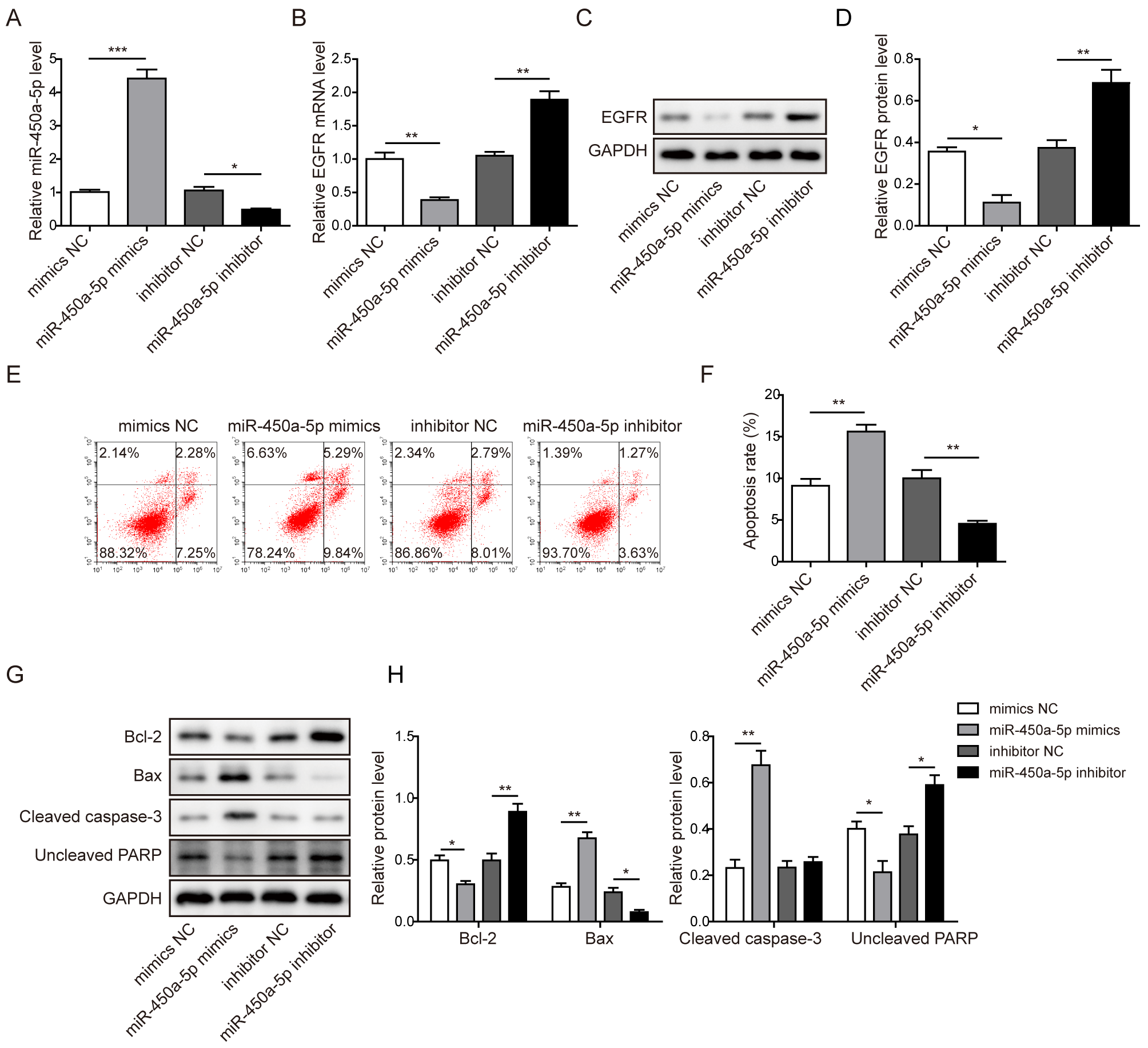

Supplement: Supplementary file 5 — Supplemental figure S2 [file 41388_2020_1422_MOESM5_ESM.tif]

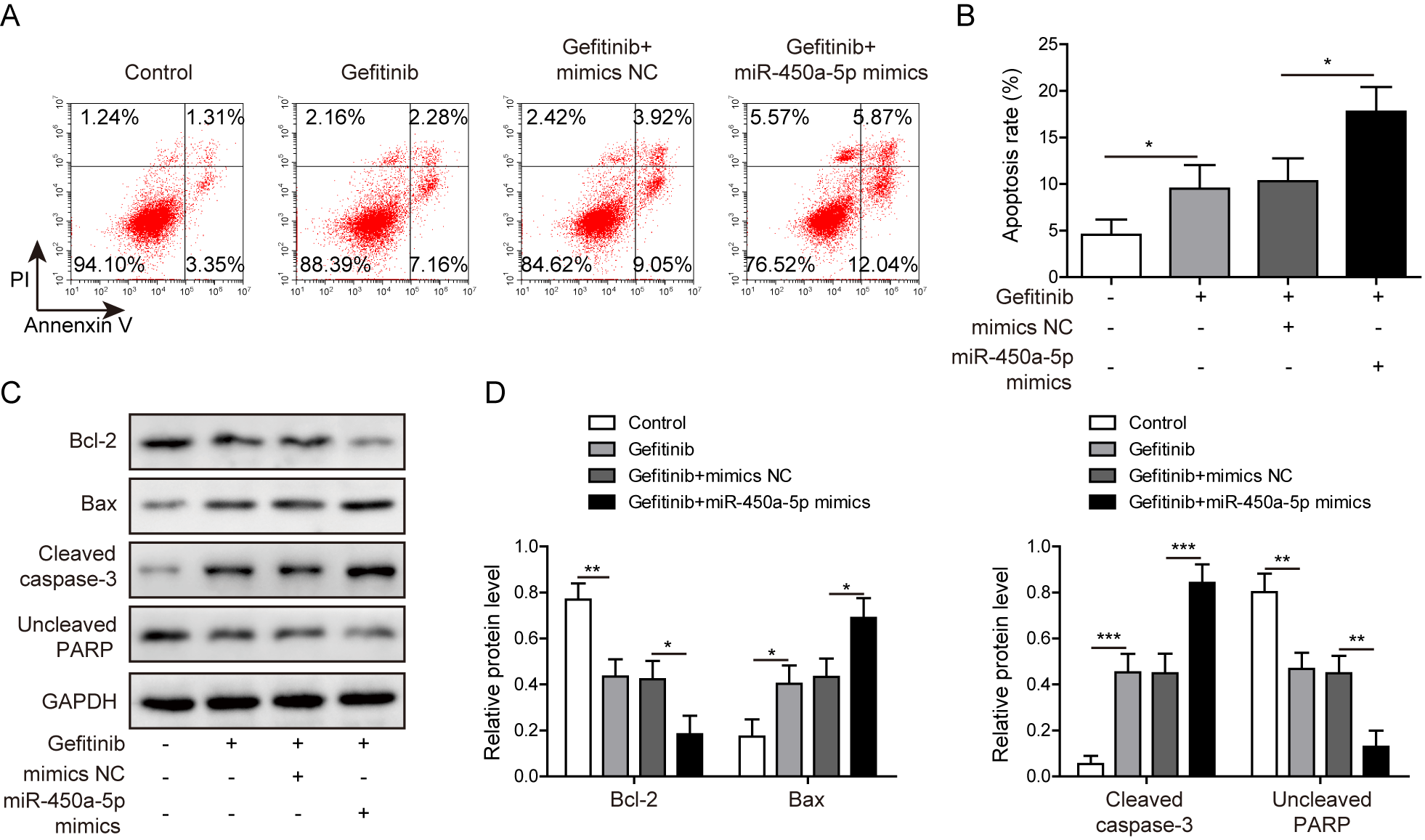

Supplement: Supplementary file 6 — Supplemental figure S3 [file 41388_2020_1422_MOESM6_ESM.tif]

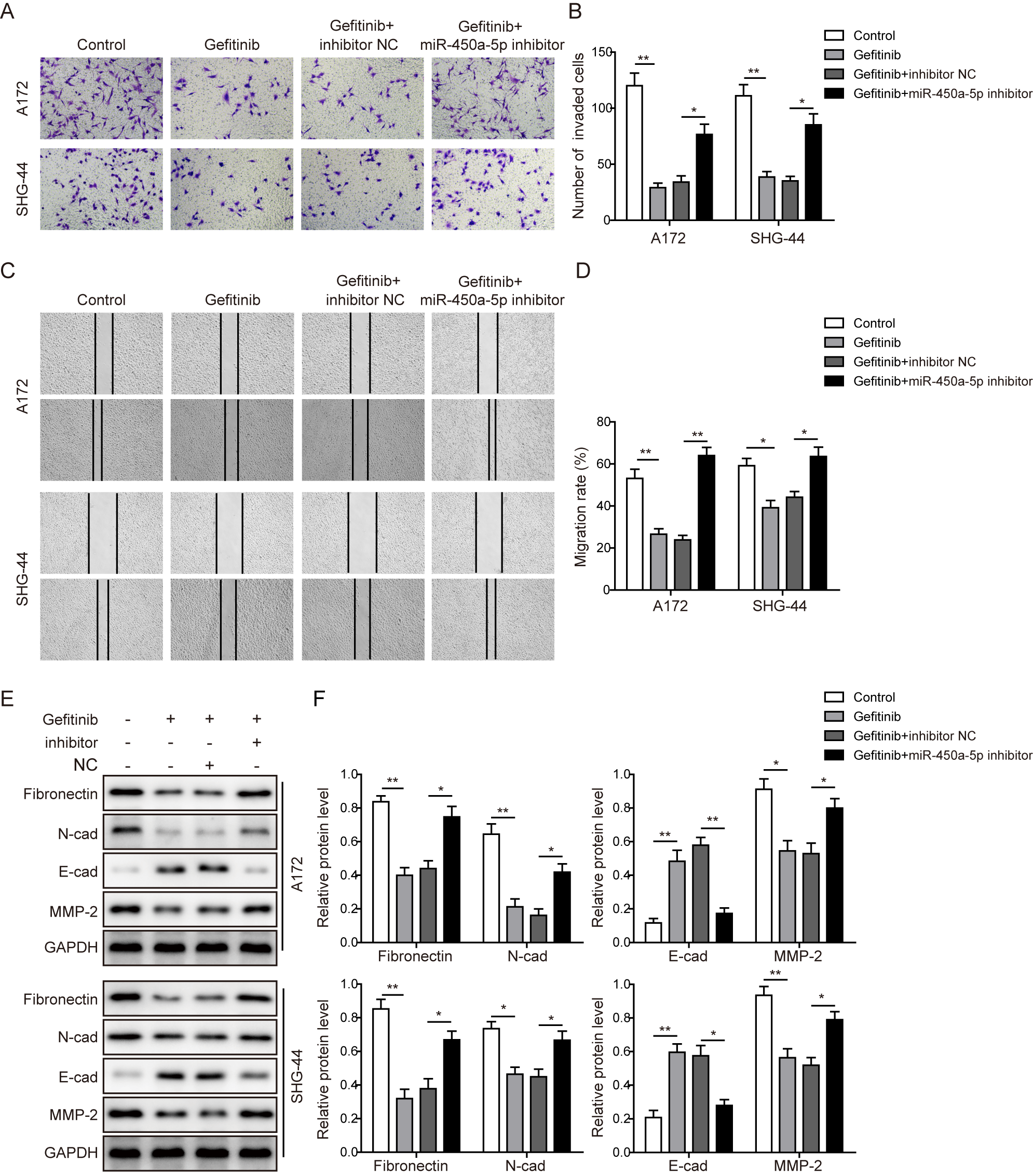

Supplement: Supplementary file 7 — Supplemental figure S4 [file 41388_2020_1422_MOESM7_ESM.tif]

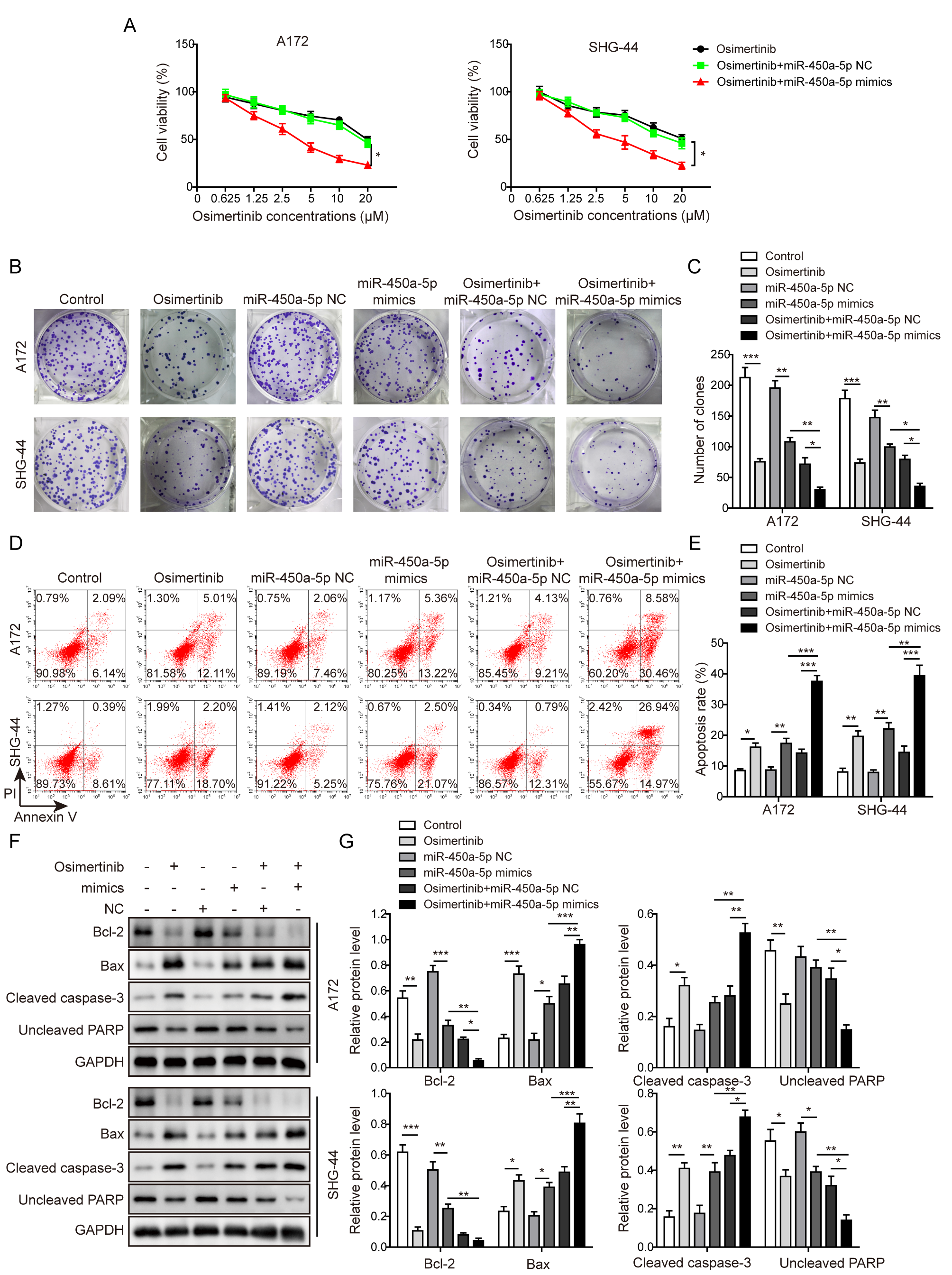

Supplement: Supplementary file 9 — Supplemental figure S5 [file 41388_2020_1422_MOESM9_ESM.tif]

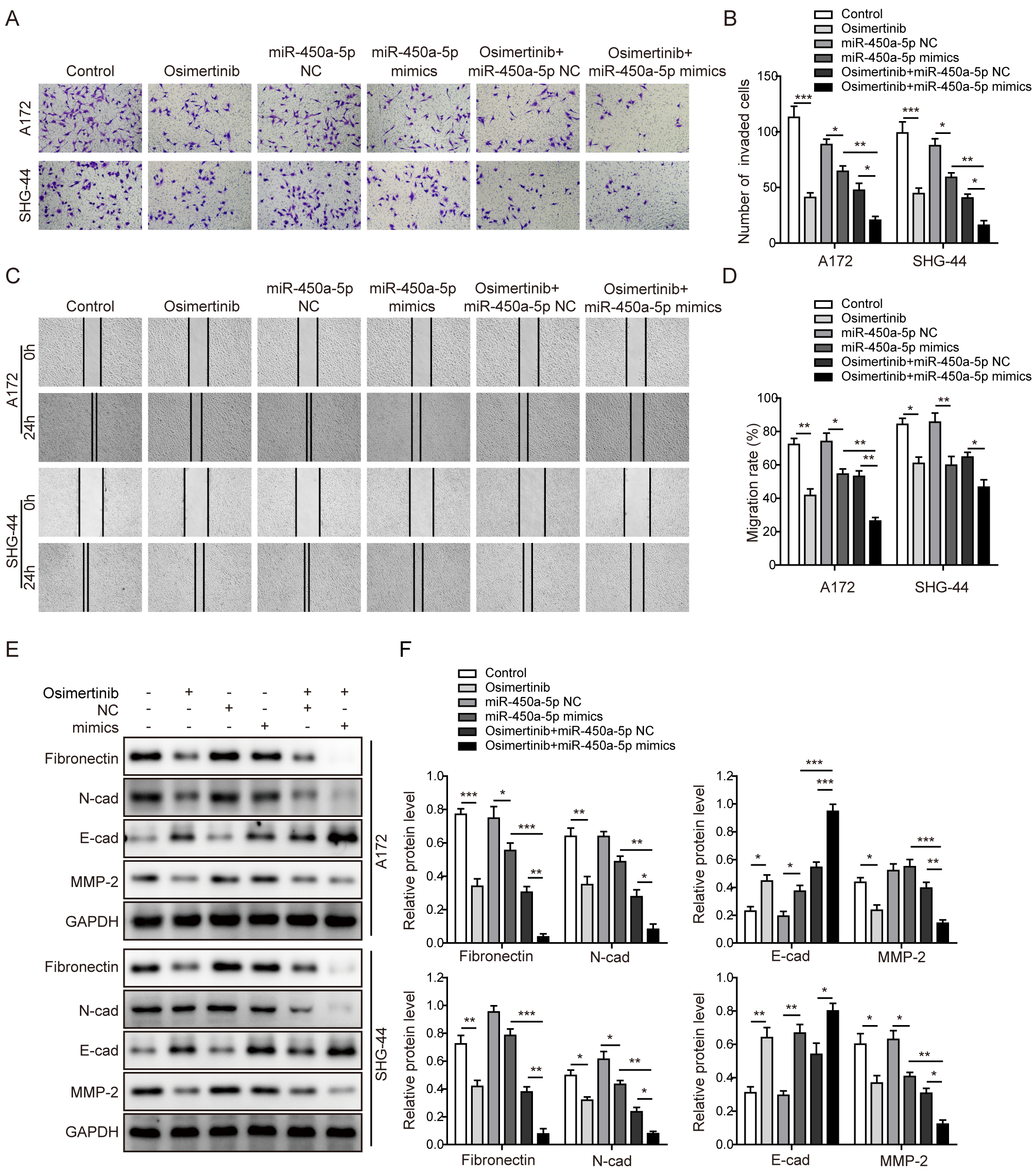

Supplement: Supplementary file 10 — Supplemental figure S6 [file 41388_2020_1422_MOESM10_ESM.tif]

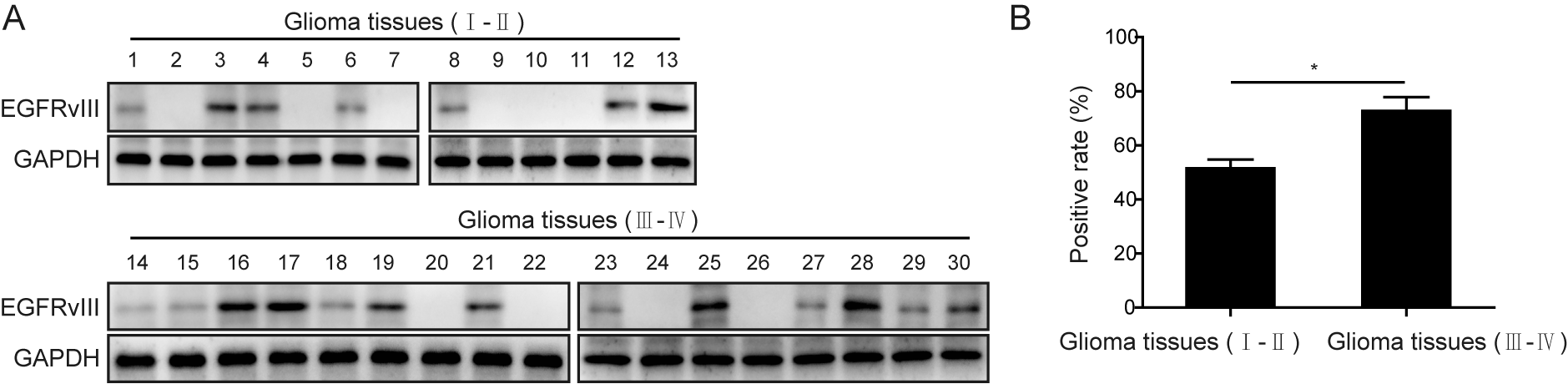

Supplement: Supplementary file 11 — Supplemental figure S7 [file 41388_2020_1422_MOESM11_ESM.tif]

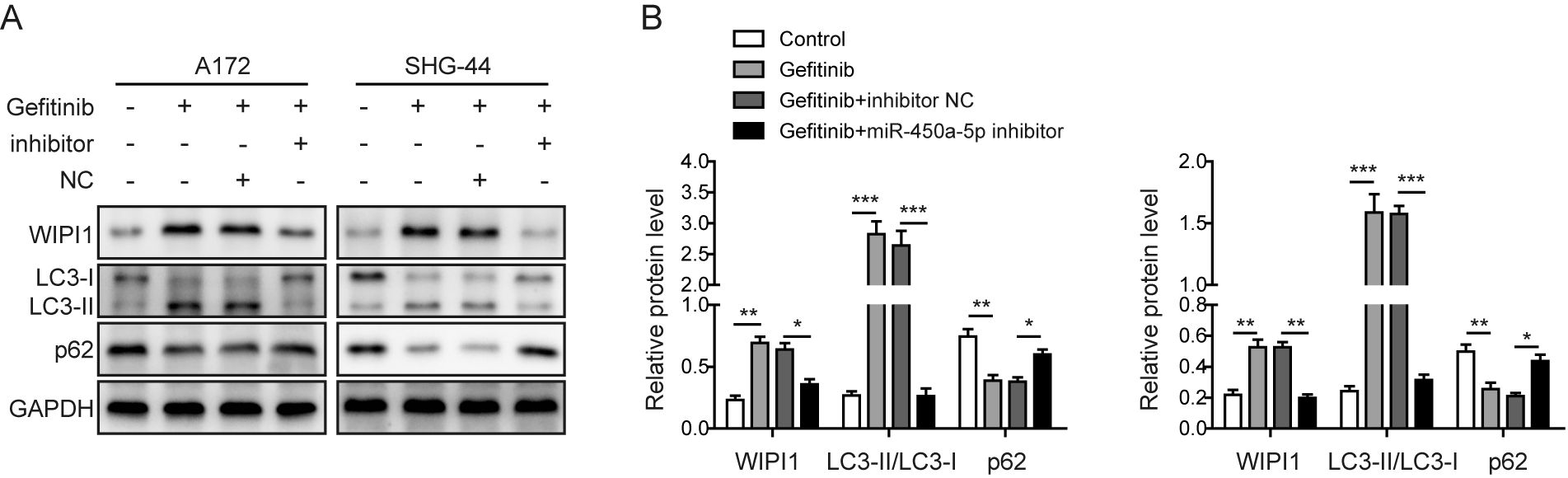

Supplement: Supplementary file 12 — Supplemental figure S8 [file 41388_2020_1422_MOESM12_ESM.tif]
