# Supplementary material for: MiR-450a-5p strengthens the drug sensitivity of gefitinib in glioma chemotherapy via regulating autophagy by targeting EGFR
Source: Oncogene. 2020 Aug 20;39(39):6190–202. doi: 10.1038/s41388-020-01422-9 (PMC7515841; doi:10.1038/s41388-020-01422-9)
Supplement: Supplementary file 8 — Supplemental Table S1 [file 41388_2020_1422_MOESM8_ESM.docx]

**Supplemental Table S1** Clinicopathological characteristics of 30 patients with glioma.

| Clinical parameters | Cases (n) | Expression level | | P value | Expression level | | P value |
| --- | --- | --- | --- | --- | --- | --- | --- |
|  |  | miR-450a-5p^high^ | miR-450a-5p^low^ | (* p<0.05) | EGFR^high^ | EGFR^low^ | (* p<0.05) |
| Age (years) |  |  |  |  |  |  |  |
| ≥60 | 18 | 11 | 7 | 0.2635 | 9 | 9 | 1.0000 |
| ＜60 | 12 | 4 | 8 |  | 6 | 6 |  |
| Gender |  |  |  |  |  |  |  |
| Female | 10 | 2 | 8 | 0.0502 | 6 | 4 | 0.6999 |
| Male | 20 | 13 | 7 |  | 9 | 11 |  |
| Smoking status |  |  |  |  |  |  |  |
| No | 18 | 8 | 10 | 0.7104 | 5 | 13 | 0.0078 |
| Yes | 12 | 7 | 5 |  | 10 | 2 |  |
| KPS score |  |  |  |  |  |  |  |
| ≤80 | 19 | 14 | 5 | 0.0017 | 9 | 10 | 1.0000 |
| ＞80 | 11 | 1 | 10 |  | 6 | 5 |  |
| WHO grade |  |  |  |  |  |  |  |
| Ⅰ-Ⅱ | 13 | 12 | 1 | 0.0001 | 3 | 10 | 0.0253 |
| Ⅲ-Ⅳ | 17 | 3 | 14 |  | 12 | 5 |  |
| Surgery |  |  |  |  |  |  |  |
| GTR | 15 | 5 | 10 | 0.1431 | 4 | 11 | 0.0268 |
| PR | 15 | 10 | 5 |  | 11 | 4 |  |
| KPS score: Karnofsky Performance Scale status score; GTR: gross total resection; PR: partial resection. | | | | | | | |
